# Supplementary material for: Transcript PHF19-207 as a Potential Biomarker for Colon Cancer Diagnosis and Screening
Source: Biomolecules. 2025 May 26;15(6):766. doi: 10.3390/biom15060766 (PMC12191053; doi:10.3390/biom15060766)
Supplement: Supplementary file 1 [file biomolecules-15-00766-s001.zip › biomolecules-3553959-supplementary.pdf]

Transcript PHF19-207 as a potential biomarker for colon cancer diagnosis and screening

Stefan Kmezic [1,4]*, Sandra Dragicevic [2], Tamara Babic [2], Jelena Ljubicic [3], Ivan Dimitrijevic [1,4], Aleksandra Nikolic [2], Velimir Markovic [1,4]

[1] Clinic for Digestive Surgery, University Clinical Center of Serbia, dr Koste Todorovica 6, 11 000 Belgrade, Serbia; kstefan1986@gmail.com (S.K); ivanclean@gmail.com (I.D); mbecmbeca@yahoo.com (V.M)
[2] Gene Regulation in Cancer Group, Institute of Molecular Genetics and Genetic Engineering, University of Belgrade, Vojvode Stepe 444a, 11042 Belgrade, Serbia; sandra.dragicevic@imgge.bg.ac.rs (S.D); tamara.babic@imgge.bg.ac.rs (T.B); aleksandra.nikolic@imgge.bg.ac.rs (A.N)
[3] Clinic for Allergy and Immunology, University Clinical Center of Serbia, dr Koste Todorovica 2, 11 000 Belgrade, Serbia; jelena.ljubicic.mfub@gmail.com (J.L)
[4] Faculty of Medicine, University of Belgrade, dr Subotica 8, 11 000 Belgrade, Serbia;
* Correspondence: kstefan1986@gmail.com; Tel.: (+381655297097)

Supplementary information

Figure S1. Schematic representation of the PHF19-207 transcript (adopted from Ensembl database).

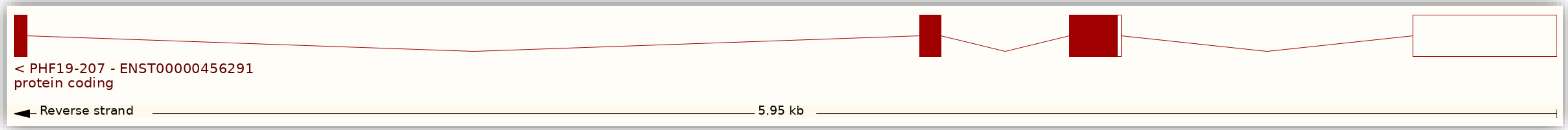

Figure S1. Schematic representation of the PHF19-207 transcript (adopted from Ensembl database).

The diagram shows the structure of the PHF19-207 transcript located on the reverse strand of chromosome 9. Coding sequences are represented as red boxes, while non-coding sequences are represented as white boxes. The lack of a complete open reading frame suggests that PHF19-207 may not produce a functional protein product.
